# Supplementary material for: Laboratory evaluation of the rapid diagnostic tests for the detection of Vibrio cholerae O1 using diarrheal samples
Source: PLoS Negl Trop Dis. 2021 Jun 15;15(6):e0009521. doi: 10.1371/journal.pntd.0009521 (PMC8232436; doi:10.1371/journal.pntd.0009521)
Supplement: S1 Table — (DOCX) [file pntd.0009521.s001.docx]

**S1 Table** Number of RDT positive samples with other pathogens identified along with

*V. cholerae* O1

| **Pathogens detected** | **No of sample** |
| --- | --- |
| *V. cholerae* O1Ogawa*, Campylobacter jejuni* | 9 |
| *V. cholerae* O1Ogawa*, C. jejuni,*  Enterotoxigenic *Escherichia coli* (ST) | 1 |
| *V .cholerae* O1Ogawa, *C. jejuni,* Enterotoxigenic *E. coli* (LT) | 1 |
| *V. cholerae* O1Ogaw*a, V. cholerae* non-O1 non-O139 | 3 |
| *V. cholerae* O1 Ogawa, Enterotoxigenic *E. coli* (ST) | 1 |
| *V .cholerae* O1Ogawa, Enterotoxigenic *E. coli* (LT) | 1 |
| *V. cholerae* O1Ogaw*a, V. cholerae* non-O1 non-O139*,* Enteroaggrigative *E. coli* | 1 |
| *V. cholerae* O1Ogawa, *Campylobacter coli* | 1 |
| *V. cholerae* O1 Ogawa*, V. fluvialis* | 2 |
| *V. cholerae* O1Ogawa*, C. jejuni, V. fluvialis* | 1 |

ST, heat-stable enterotoxin; LT, heat-labile enterotoxin
