# Supplementary material for: Laboratory evaluation of the rapid diagnostic tests for the detection of Vibrio cholerae O1 using diarrheal samples
Source: PLoS Negl Trop Dis. 2021 Jun 15;15(6):e0009521. doi: 10.1371/journal.pntd.0009521 (PMC8232436; doi:10.1371/journal.pntd.0009521)
Supplement: S2 Table — (DOCX) [file pntd.0009521.s002.docx]

**S2 Table** Duration of *V. cholerae* O1 viability in Cary-Blair medium* and RDT results after APW enrichment**

| **Assay** | **Days** | | | | | |
| --- | --- | --- | --- | --- | --- | --- |
|  | 2 | 6 | 10 | 14 | 16 | 18 |
| **Culture on TCBS** | + | + | + | + |  |  |
|  |  |  |  |  |  |  |
| **RDTs** |  |  |  |  |  |  |
| SD-Cholera | + | + | + | + |  |  |
| SMART-II | + | + | + | + |  |  |
| Crystal-VC | + | + | + | + |  |  |

*Swabs were seeded with 1 x 10^6^ CFU and stored in Cary-Blair transport medium at ambient temperature.

**APW enrichment culture was tested after 4-6 hrs.
