# Supplementary material for: Laboratory evaluation of the rapid diagnostic tests for the detection of Vibrio cholerae O1 using diarrheal samples
Source: PLoS Negl Trop Dis. 2021 Jun 15;15(6):e0009521. doi: 10.1371/journal.pntd.0009521 (PMC8232436; doi:10.1371/journal.pntd.0009521)
Supplement: Attachment — Submitted filename: Review comments.docx [file pntd.0009521.s003.docx]

Review Comments:

The manuscript entitled ‘Laboratory evaluation of the rapid diagnostic tests for the detection of Vibrio cholerae O1 using diarrheal samples’ presented data on comparative detection ability (sensitivity and specificity) of cholera rapid detection test (RDT) kits which are useful as point of care tools and of lifesaving implication. The introduction is relevant with background information, and is reasonable given the premise of the paper. The results are clear, and discussions carry sufficient information about the previous study findings. Overall, this is a good work and the data presented are important, and would be useful for relevant researchers in selecting appropriate RDT tools.

Minor comments.

The paper has weaknesses to address such as the abstract per se does not corroborate the overall results presented and needs careful rephrasing. For examples:

Page 2, Line 51-53: the sensitivity and specificity of the cholera RDT kits in the direct testing of stools was highest with SAMRT-II (86.1%) and SD-Cholera (94.4%), respectively. What are these data about, sensitivity or specificity? Can’t they present sensitivity and specificity data for all three cholera RDT kits used?

There are places that the English does not appear to be appropriate, for example:

Page 9, L 286-287: What do they mean by the sentence: ‘The results generated using the Bayesian latent class model in evaluating RDTs without the comparing the culture based “gold standard” test has been presented in Table 3?

They should address such weaknesses throughout the paper before it appears finally.
