# Supplementary material for: Laboratory evaluation of the rapid diagnostic tests for the detection of Vibrio cholerae O1 using diarrheal samples
Source: PLoS Negl Trop Dis. 2021 Jun 15;15(6):e0009521. doi: 10.1371/journal.pntd.0009521 (PMC8232436; doi:10.1371/journal.pntd.0009521)
Supplement: Attachment — Submitted filename: Response to the comments.docx [file pntd.0009521.s004.docx]

**Response to Reviewer's comments**

Key Review Criteria Required for Acceptance?
As you describe the new analyses required for acceptance, please consider the following:

**Methods**

-Are the objectives of the study clearly articulated with a clear testable hypothesis stated?
-Is the study design appropriate to address the stated objectives?
-Is the population clearly described and appropriate for the hypothesis being tested?
-Is the sample size sufficient to ensure adequate power to address the hypothesis being tested?
-Were correct statistical analysis used to support conclusions?
-Are there concerns about ethical or regulatory requirements being met?

**Reviewer #1:** Overall the methods used are adequate to achive the stated objectives, however the following points could be further clarified.
1.        It is unclear if patients with bloody diarrhea were included as part of the study. This should be clarified.

**Response:** Patients with bloody diarrhea and blood with mucus stools were also included. In the materials and methods section, we have defined diarrheal infection as passage of 3 or more loose or liquid stools in last 24 hrs or less than 3 loose/liquid stools associated with dehydration; or at least one bloody loose stool in last 24 hrs. Dysentery patients had frequent passage of stool with blood/mucus and mild to severe abdominal pain. Of the 11 samples with blood in the stools tested in this study, one was positive in the PCR assay.

We have included the above information in the results section of the revised manuscript.

2.        Authors should clarify if the Crytal VC rapid test detected both O1 and O139.

**Response:** SD Bioline Cholera and Crystal-VC can detect O1 and O139 serogroups of *V. cholerae*. We have included this information in the materials and methods section.

3.        The enriched RDT method should be described in the methods section.

**Response:** We have included the following sentence in the revised manuscript “*V. cholerae* allowed to grow in the alkaline peptone water (APW, pH 8.0) for 4 hrs from the stool specimens were also tested using the RDT kits”.

4.        A composite outcome using PCR and culture results would be more appropriate to assess the performance of the rapid test. Or otherwise the authors should conduct a separate analysis of the performance of the RDTs using PCR as the gold standard. See these reference: ([https://doi.org/10.1111/tmi.13084](https://doi.org/10.1111/tmi.13084), [https://doi.org/10.1371/journal.pone.0168257](https://doi.org/10.1371/journal.pone.0168257) ).

**Response:** We have made a separate analysis to test the performance of the RDTs using PCR as the gold standard. The results of this evaluation are presented as follows. A new table has also been included (Table 4).

We also analyzed the performance of the RDTs using PCR as the gold standard (**Table 4**). In this analysis, the sensitivity and specificity of the RDTs remained between 52.3-58.2% and 92.3-96.8%, respectively. The accuracy was low (>77%) compared to culture techniques as the gold standard. CUI with the positive predictive value was well with SMAR-II and Crystal-VC with the attribute weight ranging from 0.497 to 0.515 and all kits performed good with the negative predictive values with the attribute weights from 0.684 to 0.714. Except for SMART-II kit, comparative detection of *V. cholerae* O1 with PCR assay with the other kits is not significant (**Table 4**).

5.         It is unclear what prior were used to inform the pre-test probabilities for culture, PCR and the rapid test. This needs to be clarified.

**Response:** We have not set any prior to inform the pre-test probabilities for culture, PCR and the rapid test. All the collected samples included in the study were tested for culture (direct and enrichment), RDTs and PCR after extraction of the DNA. Before the administration of antibiotics, stool specimens were collected from the hospitalized acute diarrheal patients in the Infectious Diseases Hospital (IDH) and children treated for diarrhea as outpatients in the B. C. Roy Children Hospital (BCH), Kolkata. Samples were not considered in this study if the patients had the history of consuming antibiotics before visiting the hospital, as it may interfere with the culture results and eliminate/reduce the number of the pathogens in the stools. This study was made by fully blinding trained technicians while performing the culture of stools, RDTs and PCRs. The above information exists in the material and methods section.

6.        It will be interesting to provide estimates of the sensitivity/specificity stratified by diseases severity, age and gender.

**Response:** As suggested by this reviewer, estimates of the sensitivity/specificity stratified by disease severity, age and gender have been included as follows. This includes direct culture and PCR as gold standards. New tables are also included.

Results of sensitivity/specificity of RDTs and direct culture stratified by age, gender and diarrheal disease severity are shown in **Table 6**. Considering direct culture technique as gold standard, the sensitivity of SMART-II and Crystal VC was 100% and SD Cholera and Crystal VC were more specific (>95%) with the stool samples collected from less than 5 years age group. Among more than 5 years age group, SMART-II was more sensitive (85.2%) and SD Cholera and Crystal VC RDTs are more specific (>93%). We have analyzed RDT results of sensitivity/specificity among male and female patients. SMART-II and SD Cholera are more sensitive and specific, respectively in samples collected from both the genders (**Table 6**). In more sever cases of diarrhea, the sensitivity of all the RDTs remained the same (85%), but Crystal VC was more specific (96.6%) than the rest. Among moderate/mild cases, SMART-II and SD Cholera are more sensitive (86.2) and specific (94.3%), respectively.

Results of sensitivity/specificity of RDTs and PCR assay stratified by age, gender and diarrheal disease severity are shown in **Table 7**. Considering PCR assay as gold standard, the sensitivity of all the RDTs in less than 5 years age group remained low (≤ 38%) and SD Cholera showed more specific (97.7%). However, the sensitivity and specificity are more with SMART-II (60.8) and Crystal VC (97.1%), respectively among more than 5 years age group. SMART-II was more sensitive (56-60%) and Crystal VC are more specific (>95%) in samples collected from both the genders. In more sever cases of diarrhea, the sensitivity of all the RDT kits remained the same (52.9%), but Crystal VC remained comparatively more specific (97.8%). Among moderate/mild cases, SMART-II and Crystal VC/SD Cholera are more sensitive (59.1%) and specific (>96), respectively.

7. The sample size calculation is not provided

**Response:** The sample size for the study was based on Buderer’s formula for sensitivity and specificity of diagnostic health sciences. Results from a previous hospital study (Nair et al., 2010 [23]) reported 26% isolation of *V. cholerae* O1 from diarrhea patients of all age groups. Considering 90% specificity for the test the required sample size would be 260, for 95% confidence level and 6% absolute precision. Similarly, with 80% sensitivity of the test the required sample size would be 463. Expecting variation in the isolation proportion of the organism, we included approximately 10% more sample and finally included 506 samples in this study.

The above information has been included in the Materials and Methods section under “Sample collection”.

Reference: Nair GB, Ramamurthy T, Bhattacharya MK, Krishnan T, Ganguly S, Saha DR, et al. Emerging trends in the etiology of enteric pathogens as evidenced from an active surveillance of hospitalized diarrhoeal patients in Kolkata, India. Gut Pathog. 2010;2(1):4.

Reviewer #2: (No Response)

Reviewer #3: yes

--------------------

**Result**
-Does the analysis presented match the analysis plan?
-Are the results clearly and completely presented?
-Are the figures (Tables, Images) of sufficient quality for clarity?

Reviewer #1: Overall the results follow the analysis plan. The tables and the text support the main conclusion. Some aspects that could be improve include the following points:
1.        The analysis of the detection limit seems to indicate a lower quantity of bacteria needed from the SD rapid test to provide a positive result (6x10^7^ vs 6x10^8^). How the authors explain then the lower sensitivity of SD considering these results?

**Response:** The sensitivity of these cholera RDT kits depends on the concentration of *V. cholerae* somatic antigen (O1) antibodies. We tested the sensitivity of the kits, as this may vary between makes/brands. The detection limit of these RDT kits remained at par with other reports.

2.        The detection limit analysis and the analysis about the duration of positivity from Cary-Blair could be presented in more detail in a supplementary appendix.

**Response:** We have included a table showing the detection limit results and the duration of *V. cholerae* O1 positivity with Cary-Blair transparent medium in a supplementary table (Table S1).

3.        It would be good to include a table with the patient’s characteristics.

**Response:** In the revised manuscript, we have included a table (Table 1) showing clinical features of diarrheal patients.

Reviewer #2: (No Response)

Reviewer #3: Yes

--------------------

**Conclusions**
-Are the conclusions supported by the data presented?
-Are the limitations of analysis clearly described?
-Do the authors discuss how these data can be helpful to advance our understanding of the topic under study?
-Is public health relevance addressed?

Reviewer #1: Overall the discussion and the conclusions are supported by the results. The main limitations are described. The authors could futher improve the discussion with the following recommendations:
1.        The authors should describe in addition to outbreaks how this data is going to help to inform surveillance strategies in endemic places like India.

**Response:** The general approach for cholera diagnosis and surveillance is based on clinical examination for typical cholera symptoms and culture confirmation of stool specimens if laboratory facilities are available. Poor sampling and transportation delay of stools significantly affects the efficacy of culture methods. Considering this view, RDTs are recommended as a supplement for cholera surveillance at primary health care level. Presently, RDT is being used during outbreaks in India. Though the cholera RDTs are not considered as a diagnostic tool, but useful in the Indian Integrated Disease Surveillance Program in remote areas where the laboratory supports are not adequate.

We have included the above paragraph in the discussion section of the revised manuscript.

2.        The low specificity of PCR compare to culture is discussed. This can be the result of false negative culture results as mentioned or as well the result of PCR contamination for example. This is a very important limitation that needs to be further clarify since can widely affect the RDT performance results.

**Response:** We agree with this point and this is true when the culture based detection of *V. colore* is considered as gold standard. We have added the following sentence in the discussion section of the revised manuscript.

“The low specificity of PCR compared to culture could be the result of false negative culture results and/or the result of PCR contamination/low yield of DNA from the stools. False negative culture results may also be encountered due to the presence of low numbers of pathogens, as a consequence of antibiotic use, which in turn influence PCR results”.

3.        It is interesting to see that sensitivity values are lower than in evaluations conducted in African settings. This could indicate a lower bacterial load in the samples collected in India compared to African setting. Perhaps the authors could discuss this point referring also to other evaluations conducted in Bangladesh (another highly endemic context).

**Response:** Impact of *V. cholerae* O1 numbers in the samples and the performance of assays in different geographical areas is a very interesting but challenging question. To our knowledge, this aspect is not addressed in any study. The number of *V. cholerae* in stools depends not only on the antibiotic use, but also depend on the time of collection of stools after the onset of the disease, duration of transport, the presence of bacteria phages, etc. This aspect needs to be addressed in the future studies. We have included some of the important points in the discussion section.

4.        Providing quantification of the CFU when using culture or CT values when using quantitative PRC in different setting might help to understand this issue. Would be possible to provide a hint on this respect, specially for discordant samples (positive for culture and/or PCR and negative to RDTs)?

**Response:** The suggested point is also very important and pertinent while addressing the issues of positive for culture and/or PCR and negative to RDTs. Unfortunately, we cannot do this assay with old stool samples. Alternatively, we have included this point in the discussion section as “With respective to positive for culture and/or PCR and negative to RDTs, quantification of the *V. cholerae* CFU or CT values in quantitative PCR in discordant samples might help to understand this issue”.

5.        I guess that this lower sensitive will be even more obvious if the PCR is used as a gold standard. The authors could comment on this point as well.

**Response:** We have made a separate analysis to test the performance of the RDTs using PCR as the gold standard. The results of this evaluation are presented as follows.

We also analyzed the performance of the RDTs using PCR as the gold standard (**Table 4**). In this analysis, the sensitivity and specificity of the RDTs remained between 52.3-58.2% and 92.3-96.8%, respectively. The accuracy was low (>77%) compared to culture techniques as the gold standard. CUI with the positive predictive value was well with SMAR-II and Crystal-VC with the attribute weight ranging from 0.497 to 0.515 and all kits performed good with the negative predictive values with the attribute weights from 0.684 to 0.714. Except for SMART-II kit, comparative detection of *V. cholerae* O1 with PCR assay with the other kits is not significant (**Table 4**).

Reviewer #2: (No Response)

Reviewer #3: Yes

--------------------

**Editorial and Data Presentation Modifications?**
Use  this section for editorial suggestions as well as relatively minor modifications of existing data that would enhance clarity. If the only modifications needed are minor and/or editorial, you may wish to recommend “Minor Revision” or “Accept”.

Reviewer #1: (No Response)

Reviewer #2: (No Response)

Reviewer #3: NA

--------------------

**Summary and General Comments**

Use  this section to provide overall comments, discuss strengths/weaknesses of the study, novelty, significance, general execution and scholarship. You may also include additional comments for the author, including concerns about dual publication, research ethics, or publication ethics. If requesting major revision, please articulate the new experiments that are needed.

**Reviewer #1:**

16.        The authors should describe in addition to outbreaks how this data is going to help to inform surveillance strategies in endemic places like India.

**Response:** The general approach for cholera diagnosis and surveillance is based on clinical examination for typical cholera symptoms and culture confirmation of stool specimens if laboratory facilities are available. Poor sampling and transportation delay of stools significantly affects the efficacy of culture methods. Considering this view, RDTs are recommended as a supplement for cholera surveillance at primary health care level. Presently, RDT is being used during outbreaks in India. Though the cholera RDTs are not considered as a diagnostic tool, but useful in the Indian Integrated Disease Surveillance Program in remote areas where the laboratory supports are not adequate.

We have included the above paragraph in the discussion section of the revised manuscript.

17.        The low specificity of PCR compare to culture is discussed. This can be the result of false negative culture results as mentioned or as well the result of PCR contamination for example. This is a very important limitation that needs to be further clarify since can widely affect the RDT performance results.

**Response:** We agree with this point and this is correct when the culture based detection of *V. cholerae* is considered as gold standard. Considering this point, we have added the following sentence.

“The low specificity of PCR compared to culture could be the result of false negative culture results and/or the result of PCR contamination/low yield of DNA from the stools. False negative culture results may also be encountered due to the presence of low numbers of pathogens, as a consequence of antibiotic use, which in turn influence PCR results”.

18.        It is interesting to see that sensitivity values are lower than in evaluations conducted in African settings. This could indicate a lower bacterial load in the samples collected in India compared to African setting. Perhaps the authors could discuss this point referring also to other evaluations conducted in Bangladesh (another highly endemic context).

**Response:** Impact of *V. cholerae* O1 numbers in the samples and the performance of assays in different geographical areas is a very interesting but challenging question. To our knowledge, this aspect is not addressed in any study. The number of *V. cholerae* in stools depends not only on the antibiotic use, but also depend on the time of collection of stools after the onset of the disease, duration of transport, the presence of bacteria phages, etc. This aspect needs to be addressed in the future studies. We have included some of the important points in the discussion section.

19.        Providing quantification of the CFU when using culture or CT values when using quantitative PRC in different setting might help to understand this issue. Would be possible to provide a hint on this respect, specially for discordant samples (positive for culture and/or PCR and negative to RDTs)?

**Response:** This point is also very important and pertinent while addressing the issues of positive for culture and/or PCR and negative to RDTs. Regrettably, we cannot do this assay with old stool samples. However, we have included this point in the discussion section as follows.

“With respective to positive for culture and/or PCR and negative to RDTs, quantification of the *V. cholerae* CFU or CT values in quantitative PCR in discordant samples might help to understand this issue”.

20.        I guess that this lower sensitive will be even more obvious if the PCR is used as a gold standard. The authors could comment on this point as well.

**Response:** We have made a separate analysis to test the performance of the RDTs using PCR as the gold standard. The results of this evaluation are presented as follows.

We also analyzed the performance of the RDTs using PCR as the gold standard (**Table 4**). In this analysis, the sensitivity and specificity of the RDTs remained between 52.3-58.2% and 92.3-96.8%, respectively. The accuracy was low (>77%) compared to culture techniques as the gold standard. CUI with the positive predictive value was good with SMAR-II and Crystal-VC with the attribute weight ranging from 0.497 to 0.515 and all kits performed good with the negative predictive values with the attribute weights from 0.684 to 0.714. Except for SMART-II kit, comparative detection of *V. cholerae* O1 with PCR assay with the other kits is not significant (**Table 4**).

**Reviewer #2**: In this manuscript the authors evaluate the performance of three cholera O1 rapid diagnostic tests. The evaluation is carried out on stool samples of patients with acute diarrhoea admitted to two hospitals in Kolkata. The authors used a “classic” method for evaluation by paring the RDT result with V. choleræ culture result as gold standard. With the knowledge that the culture is an imperfect gold standard, the authors also carried out a second statistical analysis with the use of a Bayesian latent class model.
The parts of the manuscript related to the methods and the results are straight forward and well explained. There are, however, of major issues that I suggest the authors to address to. These issues are connected to each other and encompass the whole manuscript. I try here to disentangle them.

Major issues:
#1. The authors stated in the introduction (line 111) that “Cholera rapid diagnostic test (RDT) represent a promising tool in the early detection…”. Some RDT (i.e. Crystal VC) are in market for more than 10 years, and in recent years a considerable amount of research has been carried out to understand their pros and cons and the context where RDTs find their deployment. Four years ago, in the light of these works, WHO recommends the use of RDT for early detection and monitoring epidemics, but not for patient’s diagnosis.

#2. Following the statement above, and considering that the setting of this manuscript (two hospitals in Kolkata) is cholera endemic, it remains unclear the scope in which the authors frame their work on RDTs. As I said, RDTs have a clear role in surveillance and epidemic monitoring and the authors acknowledge that at the beginning of the discussion (Reference 24). In this scope, the work of this manuscript corroborates previous researches. However, if the scope is to evaluate the performance of RDTs in endemic setting, and the main purpose is the patient’s differential diagnosis (as some sentences in the introduction and in the discussion seem to indicate), the authors should clearly state that as the main purpose of their work and develop the discussion in this perspective.

**Response:** We appreciate this reviewers’ remarks. We agree that there needs some description about the purpose of this study. As mentioned by this reviewer, cholera RDT is exiting for quite some time and has undergone many modifications in order to improve the sensitivity and specificity. In this connection, we would like to highlight here that one such RDT kit i.e., Crystal VC has gone through several changes. As a consequence, the results of some investigations carried out in different settings shown different sensitivity and specificity. Testing of well known cholera RDTs in a cholera endemic setting like Kolkata has not been made in a systematic manner. Hence, we have considered three different cholera RDT kits not only evaluate their performances, but also compare the results with culture and PCR based techniques. The primary aim of the study was to determine whether the overall diagnostic performance of RDTs was equivalent to, or better than other diagnostic methods. In addition, we have made an attempt to provide estimates of the sensitivity/specificity stratified by disease severity, age and gender. Information generated from study may be useful in understanding the importance of RDT in cholera surveillance and epidemic monitoring.

In the revised manuscript, we have included the main purpose of their work in the introduction section and discussed the results in this perspective.

#3. With the main purpose of the patient’s differential diagnosis in a cholera endemic setting the use of PCR may have a role. However, I would like to point out that PCR, and its evaluation, is not mentioned anywhere in the introduction, while it is extensively mentioned in the “Material and Methods”, “Results” and “Discussion” sections. The authors even concluded the manuscript by acknowledging that “a portable PCR machine along with PCR-dipstick DNA chromatography” (line 385) may be the preferred point of care tool. This last sentence is rather confusing for two reasons: first, because this PCR variant was not evaluated in this work, and second, because the authors do not clarify in which epidemiological context the PCR-dipstick DNA chromatography would be the preferred tool. Moreover the authors should clarify why, after stating that ”PCR cannot be considered as a POC assay” (line 363), and that “PCR … cannot be used as a POC tool due to the procedural difficulties”
(lines 380-381), PCR iss eventually considered advantageous as POC test (read lines 383-384 “Considering the performance of PCR, it would be advantageous is adopting the technique for POC test”).

**Response:** To cover the PCR aspect, we have included the following sentences in the introduction section.

In addition to the culture and antibody-based RDTs, several polymerase chain reaction (PCR) techniques have been used for the rapid detection of virulence genes of *V. cholerae* [19, Nelson et al., 2020]. PCR assay is generally faster and more sensitive than the culture methods and hence identification of *V. cholerae* in stool by culture or PCR is considered the gold standard for cholera diagnosis [Alam et al., 2010; Dick et al., 2012; Page et al., 2012]. However, these assays demand suitable laboratory infrastructure, expensive equipment and skilled staff, which would not exist in remote areas where outbreaks often occur.

We agree that description about PCR-dipstick DNA chromatography might cause some confusion and hence deleted in the conclusion section of the revised manuscript.

**Reference:**

Nelson EJ, Grembi JA, Chao DL, Andrews JR, Alexandrova L, Rodriguez PH, et al. Gold Standard cholera diagnostics are tarnished by lytic bacteriophage and antibiotics. J Clin Microbiol. 2020;58(9):e00412-20. doi: 10.1128/JCM.00412-20.

Alam M, Hasan NA, Sultana M, Nair GB, Sadique A, Faruque AS, et al. Diagnostic limitations to accurate diagnosis of cholera. J Clin Microbiol. 2010;48(11):3918-22. doi: 10.1128/JCM.00616-10.

Dick MH, Guillerm M, Moussy F, Chaignat CL. Review of two decades of cholera diagnostics--how far have we really come? PLoS Negl Trop Dis. 2012;6(10):e1845. doi: 10.1371/journal.pntd.0001845.

Page AL, Alberti KP, Mondonge V, Rauzier J, Quilici ML, Guerin PJ. Evaluation of a rapid test for the diagnosis of cholera in the absence of a gold standard. PLoS One. 2012;7(5):e37360. doi: 10.1371/journal.pone.0037360.

#4.The authors should also clarify what the purpose of PCR in this work: is it used as reference-standard, as stated in the abstract and in the discussion (lines 351-352), or as a test under evaluation, as described in the results and in other parts of the discussion? The back and forth of PCR as reference-standard and as test under evaluation is a source of confusion for the readers.

**Response:** PCR was included as one of the assays to evaluate the performance of cholera RDTs. We have included the following sentence in the introduction section of the revised manuscript. “In this study, we have considered three different cholera RDT kits not only evaluate their performances, but also compare the results with culture and PCR based techniques. The primary aim of the study was to determine whether the overall diagnostic performance of RDTs was equivalent to, or better than other diagnostic methods”.

As suggested by the other reviewer, we have also done a separate analysis keeping PCR as the gold standard and included the results accordingly. To avoid confusion, we have deleted some of the overlappings PCR results from the discussion section. The 7th paragraph of the discussion section explains on the outcomes of the PCR assay.

Other minor, but not so minor, issues:
 - Abstract line 42-43: the author mention that RDTs are compared with culture and PCR method. I point out that the comparison is RDTs with PCR as reference was not presented in the manuscript.

**Response:** In the revised manuscript, we have presented the comparison is RDTs with PCR as reference

- Abstract lines 54-55: the authors mention here “After enrichment, the high sensitivity…”. At this point of the reading, there is no mention of enrichment procedure, and it not clear in which sample the enrichment was carried out. Only in the main manuscript ("Materials and Methods", "culture technique") it is mentioned that enrichment was carried out to sample before culture.

**Response:** In the abstract, we have included the following sentence in the methods section. All the stool samples were screened for the presence of *V. cholerae* by direct and enrichment culture methods”.

- Abstract lines 60-65: The conclusion is confusing as the authors seem to recommend the use of PCR, while few lines before they stated that PCR cannot be used as a point of care tool. See major issue #3 for more comments.

**Response:** We agree that description about PCR-dipstick DNA chromatography might cause some confusion and hence deleted from the abstract and conclusion sections of the revised manuscript.

- Sample collection lines 138-148: I recommend the authors to mention the dates in which the stool samples were taken.

**Response:** We have provided the following duration in which the stool samples were collected in the materials and methods section (under sample collection). Sampling was made during two consecutive peak cholera seasons, i.e., from August-December 2016 and July-November 2017.

- Data analysis line 222: “The primary endpoint … using stool culture result as the gold standard for comparison”. The authors should clarify here what results among the direct culture of the culture after enrichment is use as gold standard.

**Response:** For clarity, we have corrected the sentence as follows.

The primary endpoint is the valuation of the RDT using stool culture results, i.e. both by direct and enrichment techniques for the isolation of *V. cholerae* O1, as the gold standard for comparison.

- Discussion line 343-344: “The sensitivity of APW enrichment culture was unexpectedly low to compare direct culture method”. One effect of the APW enrichment procedure is to isolate the V. choleræ from other pathogens. So, it is not surprising that less positive sample were found in the culture samples after enrichment when compared with the direct culture where the isolation was not carried out. For this purpose, it would be interesting to compare the performances of two cultures procedures – with and without APW enrichment – with PCR as reference-standard.

**Response:** Direct stool and APW enrichment techniques as well as RDTs were used for the identification of *V. cholerae* O1. Since PCR was performed only with the DNA samples extracted from the stools, the performance PCR was compared with only direct culture method (Table 2).

**Reviewer #3:** Review comments uploaded. Accept with minor revision

Review Comments:

The manuscript entitled ‘Laboratory evaluation of the rapid diagnostic tests for the detection of Vibrio cholerae O1 using diarrheal samples’ presented data on comparative detection ability (sensitivity and specificity) of cholera rapid detection test (RDT) kits which are useful as point of care tools and of lifesaving implication. The introduction is relevant with background information, and is reasonable given the premise of the paper. The results are clear, and discussions carry sufficient information about the previous study findings. Overall, this is a good work and the data presented are important, and would be useful for relevant researchers in selecting appropriate RDT tools.

Minor comments.

The paper has weaknesses to address such as the abstract per se does not corroborate the overall results presented and needs careful rephrasing. For examples:

Page 2, Line 51-53: the sensitivity and specificity of the cholera RDT kits in the direct testing of stools was highest with SAMRT-II (86.1%) and SD-Cholera (94.4%), respectively. What are these data about, sensitivity or specificity? Can’t they present sensitivity and specificity data for all three cholera RDT kits used?

**Response:** The main constraint in the abstract section is the word limitation without going into methodological detail and summarizes the most important results with important numerical results. Considering this, we have only highlighted the main results in the abstract. In the revised manuscript, we tried to give our results as much as we could, within the word limitation.

There are places that the English does not appear to be appropriate, for example:

Page 9, L 286-287: What do they mean by the sentence: ‘The results generated using the Bayesian latent class model in evaluating RDTs without the comparing the culture based “gold standard” test has been presented in Table 3?

**Response:** For clarity, we have corrected the sentence as “Table 5 shows Bayesian latent class analysis of RDT results without the culture based “gold standard”.

They should address such weaknesses throughout the paper before it appears finally.

**Response:** We have checked the correctness of English throughout the manuscript.
