# Supplementary material for: Laboratory evaluation of the rapid diagnostic tests for the detection of Vibrio cholerae O1 using diarrheal samples
Source: PLoS Negl Trop Dis. 2021 Jun 15;15(6):e0009521. doi: 10.1371/journal.pntd.0009521 (PMC8232436; doi:10.1371/journal.pntd.0009521)
Supplement: Attachment — Submitted filename: Resonse to the Comments-2.docx [file pntd.0009521.s005.docx]

# Manuscript Title: Laboratory evaluation of the rapid diagnostic tests for the detection of *Vibrio cholerae* O1 using diarrheal samples

Reference No: **PNTD-D-20-01091R1**

1. Resposne to the Editor’s comment

(i) The authors are requested to address the comments from reviewer 2 regarding editing the original manuscript and revising the discussion. "Conversely, the authors missed the opportunity to elaborate arguments related to what is specific and innovative in this manuscript, and notably their opinion (or they strategy) in using RDTs as point of care in an endemic contex". A shorter, revised, concise manuscript will be an improvement over the current version and increase the likelihood of publication.

**Response**: We have considered this useful comment made by the reviewer-2 and revised the manuscript accordingly. We have also added a line on specific outcome of this study with reference to the use of cholera RDTs as POC tool.

(ii) We cannot make any decision about publication until we have seen the revised manuscript and your response to the reviewers' comments. Your revised manuscript is also likely to be sent to reviewers for further evaluation.

The authors are requested to address the comments from reviewer 2 regarding editing the original manuscript and revising the discussion. "Conversely, the authors missed the opportunity to elaborate arguments related to what is specific and innovative in this manuscript, and notably their opinion (or they strategy) in using RDTs as point of care in an endemic contex". A shorter, revised, concise manuscript will be an improvement over the current version and increase the likelihood of publication.

**Response:** As suggested by the Editor as well Reviewer-2, we have revised the manuscript and shortened the discussion section. We are sending the revised version for consideration.

Reviewer's Responses to Questions

Key Review Criteria Required for Acceptance?
As you describe the new analyses required for acceptance, please consider the following:

Methods
-Are the objectives of the study clearly articulated with a clear testable hypothesis stated?
-Is the study design appropriate to address the stated objectives?
-Is the population clearly described and appropriate for the hypothesis being tested?</br>
-Is the sample size sufficient to ensure adequate power to address the hypothesis being tested?
-Were correct statistical analysis used to support conclusions?
-Are there concerns about ethical or regulatory requirements being met?

Reviewer #2: (**No Response**)
--------------------

Results
-Does the analysis presented match the analysis plan?
-Are the results clearly and completely presented?
-Are the figures (Tables, Images) of sufficient quality for clarity?

Reviewer #2: (**No Response**)
--------------------

Conclusions
-Are the conclusions supported by the data presented?
-Are the limitations of analysis clearly described?
-Do the authors discuss how these data can be helpful to advance our understanding of the topic under study?
-Is public health relevance addressed?

Reviewer #2: (**No Response**)
--------------------

Editorial and Data Presentation Modifications?
Use  this section for editorial suggestions as well as relatively minor modifications of existing data that would enhance clarity. If the only modifications needed are minor and/or editorial, you may wish to recommend “Minor Revision” or “Accept”.

Reviewer #2: (**No Response**)
--------------------

**Summary and General Comments**
Use  this section to provide overall comments, discuss strengths/weaknesses of the study, novelty, significance, general execution and scholarship. You may also include additional comments for the author, including concerns about dual publication, research ethics, or publication ethics. If requesting major revision, please articulate the new experiments that are needed.

**Comment: Reviewer #2**: I acknowledge that the authors took in to account most of the suggestions proposed by the reviewers.
However, in may opinion, the discussion, despite its length, merely repeats what already said in the results section (or in the introduction) with some additional references (for example paragraph in lines 414-420 is a repetition of what written in the introduction). Conversely, the authors missed the opportunity to elaborate arguments related to what is specific and innovative in this manuscript, and notably their opinion (or they strategy) in using RDTs as point of care in an endemic context.
--------------------

**Response:** In the Discussion section, we have deleted parts of text that overlapped with the Introduction and/or Results. We have also shortened the Discussion section paying our attention to important aspects of the present findings. In addition, we expressed our view about the use of RDT as POC in cholera endemic context in the abstract, author summary, and discussion sections.
